# Utility of Serum Neopterin and Serum IL-2 Receptor Levels to Predict Absolute CD4 T Lymphocyte Count in HIV Infected Cases

**DOI:** 10.1155/2013/143648

**Published:** 2013-12-05

**Authors:** Sanjim Chadha, Preena Bhalla, Hitender Gautam, Anita Chakravarti, Sanjeev Saini, S. Anuradha, Richa Dewan

**Affiliations:** ^1^Department of Microbiology, Maulana Azad Medical College, Bahadur Shah Zafar Marg, New Delhi 110002, India; ^2^Department of Medicine, Maulana Azad Medical College, Bahadur Shah Zafar Marg, New Delhi 110002, India

## Abstract

A prospective study was carried out to evaluate the efficacy of serum neopterin and soluble IL-2 receptor (sIL-2R) concentrations in comparison to CD4 count to study the progression of HIV disease and monitor response to ART in HIV cases. One hundred newly diagnosed HIV seropositive subjects were recruited. CD4 counts were determined by FACS system. Serum neopterin and sIL-2R levels were measured using enzyme immunoassay. In our study, levels of neopterin and sIL-2R were significantly higher in subjects with CD4 <200 cells/**μ**L (with S. neopterin levels of >25.1 nmol/L and sIL-2R levels of >47.1 pM as cutoff values for CD4 <200 cells/**μ**L) compared to those in subjects with CD4 >200 cells/**μ**L at baseline which indicate that these markers can be utilized for initiation of ART in HIV cases. The levels of these markers decreased significantly after initiation of ART. In patients with CD4 >200 cells/**μ**L, these markers are helpful in predicting disease progression.

## 1. Introduction

HIV/AIDS continues to exact an enormous toll throughout the world, in both human and economic terms, posing a serious impediment to the growth and economic stability of many developing countries [1].

Infection with HIV-1 produces a prolonged, gradually progressive disease which leads to opportunistic infections and eventually death. The likelihood and timing of development of clinical AIDS following seroconversion, for any particular individual, are not readily predictable; the use of nonclinical markers has become critically important to patient management. The various phases of HIV infection, including the early asymptomatic phase, are associated with quantifiable laboratory findings. Laboratory surrogate markers of HIV infection, are by definition, measurable traits that correlate with the development of clinical AIDS [2, 3].

The cost of antiretroviral therapy has dramatically reduced and this has led to the increased use of antiretroviral therapy (ART) in developing countries. In view of that, inexpensive laboratory tests are also needed to monitor disease progression and treatment response in HIV infected individuals, living in resource-limited environments most heavily impacted by the epidemic. Currently the standard methods used to monitor HIV infection are clinical assessment, flow cytometry based CD4 T lymphocyte count (CD4 counts) measurement, and molecular assays to quantify plasma viral load (PVL). Though clinical assessment remains the most feasible approach, it lacks sensitivity in determining disease stage, progression, and therapy response; therefore, it is to be used in conjunction with laboratory measures. CD4 counts measurement and PVL quantification require expensive equipment and considerable skill, so few laboratories in resource limited countries can offer these tests free of cost. Therefore, alternative laboratory parameters are being investigated in the hope that these would reduce the economic burden of HIV/AIDS in the developing countries [4].

Chronic immune activation is a characteristic of HIV disease progression. HIV triggers polyclonal B cell activation, increased T cell turnover, production of proinflammatory cytokines, and increased number of activated T cells [5]. The levels of most cytokines in serum or plasma are not measurable in the normal state and are often not measurable in HIV infection. An alternative to measuring the excess cytokines in the circulation is the measurement of specific cytokine actions, for example, the induction of soluble products (surrogate markers for cytokines) in plasma or serum. The principal surrogate markers are neopterin, TNF receptor II (TNF-RII), CD23, C-reactive protein, and soluble interleukin 2 receptor (sIL-2R), respectively [6].

The HIV community is now faced with myriad concerns: which measures serve as the best proxies for initiation of ART, monitoring HIV disease progression, and monitoring the effect of ART and to what extent they accurately reflect patient status, the ethics of providing a particular standard of care in one area of the world but providing an alternative or substandard of care in another [7].

In view of the above, this study was undertaken to evaluate the potential role of immune activation markers including neopterin and sIL-2R levels in monitoring HIV disease among patients attending our ART clinic.

## 2. Materials and Methods

One hundred newly diagnosed HIV seropositive subjects were enrolled for the study after obtaining an informed consent and proper counseling. The subjects were registered with the ART clinic of the hospital. Subjects were then referred to CD4 testing.

At the time of recruitment, blood samples were collected for CD4 counts, serum IL-2R, and serum neopterin. All study subjects were categorized into three study groups: CD4 counts <200 cells/*μ*L (group A, 33 subjects), 200–500 cells/*μ*L (group B, 34 subjects), and >500 cells/*μ*L (group C, 33 subjects). Blood sample for PVL estimation was obtained for group A subjects at recruitment.

CD4 counts were determined by the FACS system (Becton Dickinson). The levels of serum neopterin were measured using the commercially available enzyme immunoassay (B.R.A.H.M.S). The levels of soluble interleukin 2 receptor (sIL-2R) were measured by the commercially available enzyme immunoassay (Immunotech). Plasma viral load (PVL) was estimated using Amplicor HIV-1 Monitor Test, version 1.5 (Roche Diagnostics), by the standard procedure.

Group A cases were initiated on ART including 2 nucleoside reverse transcriptase inhibitors (NRTIs) plus 1 nonnucleoside reverse transcriptase inhibitors (NNRTI) as per National AIDS Control Organisation recommendations [8]. These cases were followed up after six months for evaluation of response to ART by measuring the CD4 counts, levels of sIL2R, serum neopterin, and PVL.

Groups B and C cases were followed up after a period of six months to monitor disease progression by measuring the CD4 counts, levels of sIL2R, and serum neopterin.

Forty-two age and sex matched HIV negative healthy controls were also included for comparing CD4 counts, levels of sIL2R, and serum neopterin with HIV positive individuals.

To detect whether there was a significant difference in the levels of individual markers between baselines and followup wilcoxon signed rank test was applied. To analyze the correlation between different markers at baseline and at followup Spearman's rank correlation test was applied. SPSS software was used for analyzing the data. The diagnostic performance of different immunological tests was evaluated using receiver operating characteristic (ROC) curve analysis.

## 3. Results

Median age of HIV seropositive study subjects was 32.5 years. The predominant age group in males and females was 26–30 years. The majority (75%) of the study subjects enrolled were from urban areas. Thirty-one percent males and forty-two percent females were illiterate (Table 1).

The values of CD4 counts were significantly higher (*P* < 0.5) in the healthy control group as compared to HIV positive study subjects (805 cells/*μ*L versus 279 cells/*μ*L) at baseline. The values of neopterin (6.3 nmol/L versus 25.5 nmol/L) and sIL-2R (4.4 pM versus 63.0 pM) were significantly lower (*P* < 0.5) in the healthy control group as compared to the HIV seropositive study subjects (Table 1).

Out of the 100 subjects recruited in our study only 68 subjects reported for followup. Nine subjects expired and 23 were lost to followup.

Among group A subjects, adherence rate was >95%. Six (18.2%) subjects died within six months and 5 (15.2%) were defaulters. Twenty two (66.6%) subjects reported for followup. Among group B subjects, one subject (2.9%) died within six months and 3 subjects (8.8%) were defaulters. Thirty (88.2%) subjects reported for followup. Among group C subjects, two subjects (6.1%) died within six months and 15 subjects (45.4%) were defaulters. Sixteen (48.5%) subjects reported for followup.

Among group A, there was a statistically significant rise in the median CD4 counts after ART although two cases showed immunological failure as per NACO guidelines. There was statistically significant decrease in median neopterin levels and median level of sIL-2R after followup. At baseline, PVL ranged from 2636 to >750,000 copies/mL with a median PVL at the baseline of 165,000 copies/mL. At followup 90% of study subjects had undetectable level of viremia (viral load of <400 copies/mL), while 2 cases showed 407 & 1100 copies/mL of HIV-1 RNA. Among group B, there was a fall in the level of CD4 counts at followup which was statistically insignificant but a statistically significant increase in median level of neopterin and median level of sIL-2R at followup was observed. Among group C, a statistically significant fall in the CD4 counts was observed at followup along with the statistically significant increase in median level of neopterin and median level of sIL-2R at followup (Table 2).

Among group A, CD4 count had a negative correlation with neopterin and sIL-2R at baseline and followup both but it was statistically insignificant. PVL had a positive correlation with serum neopterin (correlation coefficient: 0.375, *P* value: 0.103) and sIL-2R (correlation coefficient: 0.517, *P* value; 0.020) at baseline. Among group B, CD4 count had a negative correlation with serum neopterin and sIL-2R at baseline and followup both and it was found to be statistically significant only for neopterin at followup. Among group C, CD4 count had a negative correlation with neopterin and sIL-2R at baseline and followup both, but it was statistically insignificant (Table 3).

Median levels of serum neopterin (38.6 nmol/L versus 19.5 nmol/L, *P* value <0.5) and sIL-2R (96.1 pM versus 46.6 pM, *P* value <0.5) were significantly higher in subjects with CD4 counts <200 cells/*μ*L as compared to subjects with CD4 counts >200 cells/*μ*L.

In our study, ROC curve analysis suggests S. neopterin levels of >25.1 nmol/L and sIL-2R levels of >47.1 pM as cut off value for CD4 count of <200 cells/*μ*L (Table 4).

## 4. Discussion

All group A subjects in our study showed good clinical improvement after six months of ART and no subject showed clinical failure. There was a statistically significant rise (111 cells/*μ*L to 216 cells/*μ*L) in the CD4 counts among subjects on ART, indicating a good immunological response which is supported by another Indian study, in which the median CD4 cell count after 3 months of ART rose to 304 cells/mm^3^, and at 6 months the median was 328 cells/mm^3^ [9]. For groups B and C subjects, the median CD4 counts at baseline were 305 cells/*μ*L & 580 cells/*μ*L, while at followup the median count was 274 cells/*μ*L & 487 cells/*μ*L, respectively. In a study conducted by Mehendale et al. on Indian seroconverters, between the two periods of 91–360 days after seroconversion and 361–720 days after seroconversion, median CD4 counts dropped from 631/mm^3^ to 497/mm^3^. The annual decline in CD4 cell count was 120 cells/year among patients who were not on ART [10]. A study from France demonstrated that the difference between baseline CD4 T-cell count and count after 3 years was −240/mm^3^ in the untreated group of HIV-1 infected patients with CD4 T-cell count above 500/mm^3^ [11].

Among group A subjects, ART significantly decreased the levels of S. neopterin (38.6 nmol/L to 11.2 nmol/L) after six months of ART (70.9% reduction). Our results correlate with a study conducted by Amirayan-Chevillard et al. which showed that ART significantly decreased the circulating levels of neopterin by about 30% (15.6 ± 1.5 ng/mL for treated versus 22.3 ± 3.7 ng/mL for naïve patients) [12]. For groups B and C subjects, the median level of neopterin at baseline was 22.9 nmol/L and 16.2 nmol/L, respectively, which significantly increased to 37 nmol/L and 27.6 nmol/L at followup, respectively. In a study by Mildvan et al. in 2005 it was shown that elevated baseline values for neopterin were associated with greater subsequent risk of clinical disease progression. In a head-to-head comparison that was adjusted for CD4 counts and HIV-1 RNA levels, elevated values for neopterin were the strongest predictors of increased risk of clinical disease progression 6 months later [13]. In another study by Mellors et al., it was seen that plasma viral load was the single best predictor of progression to AIDS and death, followed by CD4^+^ lymphocyte count and serum neopterin levels [14].

Among group A, the median level of sIL-2R significantly decreased (*P* value <0.5) from 96.1 pM to 45.5 pM after six months of ART. Our findings were in contrast to the findings of Bonnet et al., who in their study established that sIL-2R levels did not decrease significantly throughout the follow-up period in 46 therapy naïve patients (difference of −0.32 IU/mL, *P* value: 0.44 between baseline and a followup after 12 months of ART) [15]. There was a significant rise in the level of sIL-2R for groups B & C subjects from 60.9 pM & 35.1 pM at baseline to 77.8 pM & 53.1 pM at followup. Schulte and Meurer in their study measured sIL-2R levels in 88 patients with HIV infection. Mean sIL-2R values increased with the progression of the disease and were most pronounced in patients with AIDS. They established that sIL-2R is a valuable parameter in monitoring the course of the HIV infection [16].

In our study, among group A, 90% of study subjects at followup had undetectable level of viremia (PVL of <400 copies/mL) which is supported by another study in which 95% of these patients had PVL less than 400 copies/mL after 6 months of ART [9]. PVL had a positive correlation with neopterin and sIL-2R at baseline but this correlation was significant for only sIL-2R (*ρ* = 0.517, *P* value <0.05). In one earlier study no correlation was found between evolutions of HIV RNA and sIL-2R from baseline to six months after ART (*P* > 0.05), but a significant positive correlation was noted between evolution of sIL-2R and HIV RNA (*r* = 0.36, *P* < 0.05) from baseline to month 12 [15].

Median levels of serum neopterin & sIL-2R were found to be significantly higher in subjects with CD4 counts <200 cells/*μ*L as compared to subjects with CD4 counts >200 cells/*μ*L (*P* value <0.05). In our study, ROC curve analysis suggests S. neopterin levels of >25.1 nmol/L (sensitivity 90.9%, specificity 67.4%) and sIL-2R levels of >47.1 pM (sensitivity 95.5%, specificity 60.9%) as cutoff values for CD4 count of <200 cells/*μ*L. This observation suggests that these markers can be utilized for initiation of antiretroviral therapy in HIV infected individuals. However, their cutoff values need to be calculated from further larger studies.

Levels of these markers also showed significant decreases in response to ART at followup. However, larger studies are required to derive the levels of these markers that suggest an appropriate treatment response. In patients with CD4 counts >200 cells/*μ*L, these markers are helpful in predicting disease progression; however, large scale studies are required to establish the utility of these markers in predicting the disease progression.

## Figures and Tables

**Table 1:** Demographic and baseline characteristics of the study subjects.

Characteristic	Group A (CD4 < 200) (*n* = 33)	Group B (CD4 200–500) (*n* = 34)	Group C (CD4 > 500) (*n* = 33)	HIV positive subjects (*n* = 100)	Healthy control group (*n* = 42)
Median (IQR) of age in yrs	32.0 (25–41)	32.5 (27–39)	33.0 (24–39)	32.5 (24–41)	31.5 (26–40)
Males/females (No.)	22/11	23/11	22/11	67/33	28/14
Marital status					
Married	23	26	22	71	30
Unmarried	6	5	7	18	9
Widowed	4	3	4	11	3
Risk behaviour					
Heterosexual	30	29	27	86	38
IVDU*	2	4	3	9	4
MSM	1	0	2	3	0
Not known	0	1	1	2	0
Median (IQR) CD4 count (cells/*µ*L)	111 (79–154)	305 (259–392)	580 (524–750)	279 (154–482)	805 (645–1063)
Median (IQR) of S. neopterin (nmol/L)	38.57 (25.7–126.0)	22.9 (13.4–40.3)	16.24 (10.3–23)	25.5 (15.6–39.7)	6.3 (4.7–6.8)
Median (IQR) of sIL-2R (pM)	96.09 (61.4–139.0)	60.85 (40.8–86.8)	35.09 (25.2–64.6)	63 (42.6–100.1)	4.4 (1.9–8.1)
Mean (range) of PVL (copies/mL)	145,000 (2436–>750,000)	ND^#^	ND	145,000 (2436–>750,000) *n* = 33, group A only	ND

*Intravenous drug users.

^#^ND: not done.

**Table 2:** Change in the level of markers from baseline to followup in the three groups.

Marker	Group A (CD4 < 200)			Group B (200–500)			Group C (>500)		
	Baseline Median (IQR)	FU Median (IQR)	*P* value	Baseline Median (IQR)	FU Median (IQR)	*P* value	Baseline Median (IQR)	FU Median (IQR)	*P* value
CD4 count (cells/*µ*L)	111 (79–154)	216 (159–253)	0.000	305 (259–392)	274 (200–406)	0.162	580 (524–750)	487 (383–576)	0.008
S. neopterin (nmol/L)	38.6 (27.5–128.1)	11.2 (8.13–21)	0.00	22.9 (13.4–40.3)	37.1 (16.6–63.3)	0.003	16.2 (10.3–23)	27.6 (19.2–43.1)	0.001
sIL-2R (pM)	96.1 (61.4–139.1)	45.5 (26.7–65.2)	0.000	60.9 (40.9–86.8)	77.8 (56.9–98.2)	0.017	35.1 (25.2–64.6)	53.1 (37.5–84.9)	0.004

**Table 3:** Correlation of neopterin and sIL-2R with CD4 count at baseline and followup in the three groups.

Marker	Group A (CD4 < 200) (*n* = 22)		Group B (CD4 200–500) (*n* = 30)		Group C (CD4 > 500) (*n* = 16)	
	Baseline *ρ* (*P* value)	FU *ρ* (*P* value)	Baseline *ρ* (*P* value)	FU *ρ* (*P* value)	Baseline *ρ* (*P* value)	FU *ρ* (*P* value)
S. neopterin	−0.021 (0.924)	−0.160 (0.477)	−0.018 (0.924)	−0.669 (0.000)	−0.122 (0.652)	−0.366 (0.163)
sIL-2R	−0.165 (0.463)	−0.158 (0.484)	−0.137 (0.472)	−0.356 (0.054)	−0.376 (0.152)	−0.350 (0.184)

*ρ*: correlation coefficient (Spearman's test).

**Table 4:** Measures of validity and roc curve area of neopterin and sIL-2R in relation to the CD4 cell count <200 cells/*μ*L.

Measures of validity	S. neopterin (95% CI)	sIL-2R (95% CI)
Cutoff value	>25.1 nmol/L	>47.1 pM
Sensitivity	90.9 (95% CI: 70.8–98.9)	95.5 (95% CI: 77.2–99.9)
Specificity	67.4 (95% CI: 52.0–80.5)	60.9 (95% CI: 45.4–74.9)
Positive predictive value	86.4 (74.5–93.2)	92.3 (79.1–96.4)
Negative predictive values	70.0 (61.3–78.8)	58.7 (49.3–72.8)
ROC curve area	0.83 (0.71–0.90), *P* = 0.0001	0.82 (0.71–0.90), *P* = 0.0001

## References

[1] Fauci AS (1999). The AIDS epidemic—considerations for the 21st century. *The New England Journal of Medicine*.

[2] Pascale JM, Isaacs MD, Contreras P (1997). Immunological markers of disease progression in patients infected with the human immunodeficiency virus. *Clinical and Diagnostic Laboratory Immunology*.

[3] Tsoukas CM, Bernard NF (1994). Markers predicting progression of human immunodeficiency virus-related disease. *Clinical Microbiology Reviews*.

[4] Balakrishnan P, Solomon S, Kumarasamy N, Mayer KH (2005). Low-cost monitoring of HIV infected individuals on highly active antiretroviral therapy (HAART) in developing countries. *Indian Journal of Medical Research*.

[5] Langford SE, Ananworanich J, Cooper DA (2007). Predictors of disease progression in HIV infection: a review. *AIDS Research and Therapy*.

[6] Fahey JL (1998). Cytokines, plasma immune activation markers, and clinically relevant surrogate markers in human immunodeficiency virus infection. *Clinical and Diagnostic Laboratory Immunology*.

[7] Kimmel AD (2006). *Monitoring Disease Progression in HIV-Infected Individuals—Considerations for Developed and Developing Country Contexts*.

[8] WHO (2007). *Anti-Retroviral Therapy Guidelines for HIV-Infected Adults and Adolescents Including Post-Exposure Prophylaxis*.

[9] Kumarasamy N, Vallabhaneni S, Flanigan TP (2005). Rapid viral load suppression following generic highly active antiretroviral therapy in southern Indian HIV-infected patients. *AIDS*.

[10] Mehendale SM, Risbud AR, Thakar MA (2002). Rapid disease progression in human immunodeficiency virus type 1-infected seroconverters in India. *AIDS Research and Human Retroviruses*.

[11] Piroth L, Binquet C, Buisson M (2004). Clinical, immunological and virological evolution in patients with CD4 T-cell count above 500/mm3: is there a benefit to treat with highly active antiretroviral therapy (HAART)?. *European Journal of Epidemiology*.

[12] Amirayan-Chevillard N, Tissot-Dupont H, Obadia Y, Gallais H, Mege J-L, Capo C (2000). Highly active antiretroviral therapy (HAART) and circulating markers of immune activation: specific effect of HAART on Neopterin. *Clinical and Diagnostic Laboratory Immunology*.

[13] Mildvan D, Spritzler J, Grossberg SE (2005). Serum neopterin, an immune activation marker, independently predicts disease progression in advanced HIV-1 infection. *Clinical Infectious Diseases*.

[14] Mellors JW, Muñoz A, Giorgi JV (1997). Plasma viral load and CD4^+^ lymphocytes as prognostic markers of HIV-1 infection. *Annals of Internal Medicine*.

[15] Bonnet F, Savès M, Morlat P (2002). Correlations of soluble interleukin-2 and tumor necrosis factor type II receptors with immunologic and virologic responses under HAART. *Journal of Clinical Immunology*.

[16] Schulte C, Meurer M (1989). Soluble Il-2 receptor serum levels—a marker for disease progression in patients with HIV-1 infection. *Archives of Dermatological Research*.

